# Decreased Temperature Facilitates Short-Term Sea Star Wasting Disease Survival in the Keystone Intertidal Sea Star *Pisaster ochraceus*

**DOI:** 10.1371/journal.pone.0153670

**Published:** 2016-04-29

**Authors:** Arielle W. Kohl, Timothy I. McClure, Benjamin G. Miner

**Affiliations:** Western Washington University, 516 High Street, Bellingham, WA, 98225, United States of America; US Geological Survey, UNITED STATES

## Abstract

An extensive 2013 mass mortality event along the West Coast of North America due to Sea Star Wasting Disease (SSWD) has affected at least 20 species of sea stars. Among environmental factors potentially contributing to the timing of the current outbreak, increased coastal water temperatures are hypothesized to have contributed to previous and current outbreaks of SSWD. With a laboratory experiment, we tested whether cooler temperatures, similar to average winter temperatures, compared to average summer temperatures could slow the progression of morbidity or prevent SSWD mortality entirely in *Pisaster ochraceus*. Sea stars housed in cooler water progressed through SSWD states more slowly than sea stars housed at summer temperatures. However, the cooler temperature did not prevent SSWD mortality, and all stars died of the disease. Our data are consistent with experimental studies and field observations during previous and current outbreaks, and support the hypothesis that changes in coastal water temperatures have influenced one of the largest disease related mass mortality events in our oceans.

## Introduction

Sea star wasting disease (SSWD) has recently devastated populations of sea stars along the West Coast of North America. In the summer of 2013 along the coasts of British Columbia, California, and Washington, a large-scale outbreak of SSWD produced mass die-offs of the sunflower star *Pycnopodia helianthoides* Brandt 1835, and die-offs of other species soon followed [1]. To date, the current outbreak encompasses the entire Pacific coast of North America, from Baja California, MX to Alaska, USA and has impacted at least 20 known species and several genera [1]. However, the impact of SSWD does appear to differ among species [pers. obs.]. For example, the well-known ochre star, *Pisaster ochraceus* Brandt 1835 was, prior to 2013, very common along the West coast of North America, but is now uncommon or absent from most sites within its’ former range [1]. In contrast to *P*. *ochraceus*, the blood star, a species complex of the genus *Henricia*, is still common throughout the Salish Sea [pers. obs.]. Despite this variability, populations of many previously common species are greatly reduced or absent in regions where they were once common [1], and SSWD has caused one of the most extensive marine mass-mortality events observed.

**Fig 1:**
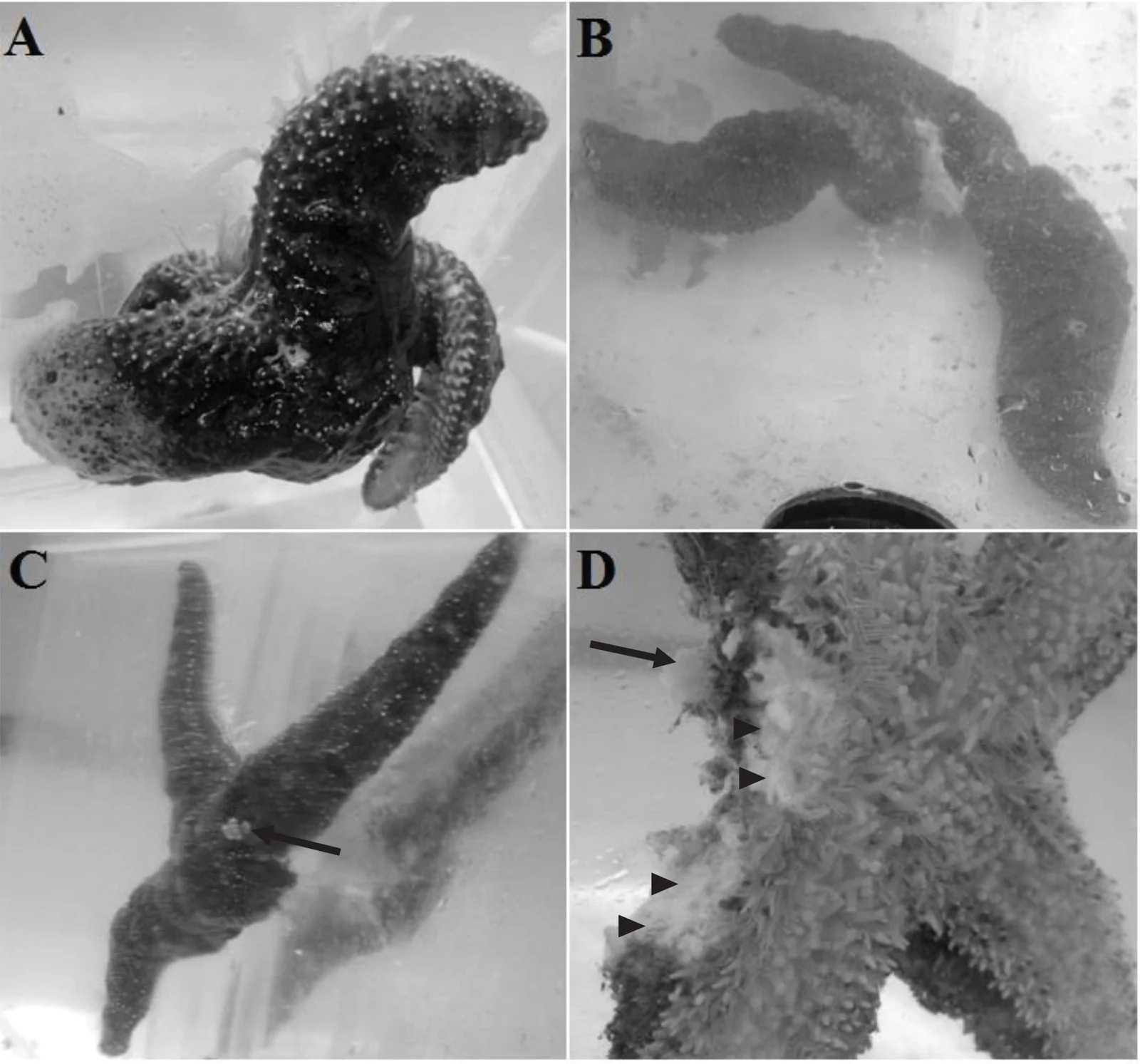
Individuals of *P*. *ochraceus* with signs of SSWD. (A) Typical arm twisting behavior involved contortions of one or more arms toward the oral disk, often observed prior to lesion spread or development. (B) ‘Corkscrew’ arm twisting, contortions were generally limited to one arm and involved the twisting of the affected arm about a central axis, and often observed immediately prior to shedding of the affected arm. (C) Shedding of one or more arms generally occurred 1–5 days after observing substantial lesioning; several individuals displayed perforated aboral lesions with protruding pyloric cecae or gonads (arrow). (D) Close-up of the wound produced by a recent arm-shedding event, solid masses of lesioned tissue (arrowheads) often appeared to close or occlude openings in the coelomic cavity, though gonads and pyloric cecae (arrow) were often observed to protrude through wounds produced by arm shedding.

SSWD is characterized by a progression of observable signs [2]. Initially, sea stars are lethargic and disinterested in feeding, even when given access to plenty of prey. These initial symptoms are followed by loss of body turgor, contortion or twisting of arm rays over the oral disc, appearance of white lesions in the epidermis and body wall, autotomy of rays, and ultimately death (Fig 1). Dead individuals rapidly disintegrate, producing white piles of ossicles and decomposing tissue, hallmarks of SSWD mortality; however the rate of progression appears to differ slightly among species [1].

Although the cause of SSWD is not yet known, there is evidence that both viral and bacterial infections might be involved in the disease [2]. During several previous outbreaks, investigators found bacteria in the genus *Vibrio* in autopsied specimens [3, 4, 5]. Increased transcription of bacterial genes from *Pseudomonas* and *Vibrio* species were also observed in infected *P*. *helianthoides* [6]. However, these bacteria might be the result of secondary infections, and not the root cause of SSWD. Currently the best candidate for a root cause of SSWD is a marine virus; sea star-associated densovirus (SSaDV), identified with metagenomic techniques [2]. SSaDV appears to be capable of producing SSWD symptoms and mortality; however it has been isolated from both asymptomatic field-collected animals, and 72y old, asymptomatic museum specimens [2]. This raises several pressing questions concerning the timing of the current mass mortality event. If SSaDV has been present in detectable levels for almost three quarters of a century, the sudden, massive scale of the current mortality event with little apparent historical precedent is perplexing. Possible explanations for the timing and severity of the current outbreak might lie with recent variations in environmental factors. Among these factors, increases in water temperatures might have a particularly large influence on these ectothermic organisms, and thus on the mortality of sea stars due to SSWD.

Local water temperature has increased preceding or during the appearance of SSWD in previous, localized outbreaks [4, 5, 7]. Short exposures (96 hours) to temperature increases as small as 4°C were sufficient to induce SSWD-like symptoms in healthy *P*. *ochraceus* in Barkley Sound, British Columbia [3]. In field surveys conducted during the summer of 2008, infections of SSWD occurred more consistently in the month of August, with recorded water temperatures of approximately 14°C, than two months earlier in June when water was cooler [3]. Increases in water temperature preceding the appearance of SSWD were also observed during a mass mortality event of *Heliaster kubiniji* Xantus 1860 in the Gulf of California in the summer of 1978 [7]. In the Channel Islands off the coast of California, smaller scale wasting and mass mortality events (with infection documented in approximately 10 Asteroid, 3 Echinoid, and one Holothuroid species) were observed in association with warmer water temperatures in 1978, and again in 1982–3 and 1997 during El Niño events [4, 7]. During the most recent SSWD outbreak, we have also observed many more sick and dead stars in the Salish Sea during periods of warmer water from June through early September than during the winter and early spring months when temperatures are cooler [pers. obs.].

There is also recent evidence that temperature has influenced the recent outbreak of SSWD [8]. Consistent with studies from previous SSWD events [3, 5], warmer temperatures were correlated with SSWD mortality within the Salish Sea, and individuals reared in warmer water were more likely to die of SSWD [8]. The experimental evidence is compelling but confounds warmer temperatures with greater changes in temperatures [3, 5, 8]. In these experiments individuals were acclimated to the coolest temperatures of the experiment. Thus, individuals in warmer treatments were also exposed to the greatest change in temperature, which could physiologically stress an individual, making them more susceptible to SSWD. It is therefore difficult to know whether warmer temperatures *per se*, or greater changes in temperature explain the results.

In this study, we hypothesized that temperature influences the susceptibility of the sea star *P*. *ochraceus* to SSWD. However, rather than testing whether warmer temperatures increased the risk of the healthy stars developing signs of SSWD (similar to [3, 5, 8]), we tested whether cooler temperatures could slow or reverse the progression of SSWD in infected individuals. With this design, we can also test whether warmer temperatures *per se* or greater changes in temperature affect SSWD morbidity and mortality. *Pisaster ochraceus* is an intertidal and shallow subtidal species that can strongly influence the structure of nearshore communities [9], and has been heavily impacted by SSWD throughout its range [1].

## Materials and Methods

We designed a laboratory experiment to determine if cooler temperatures might slow SSWD progression or prevent mortality due to SSWD. To test our hypothesis, we collected *P*. *ochraceus* during the summer and housed some individuals in water heated to ambient local summer temperatures, while the other stars were placed in water chilled to local winter temperatures. We then monitored SSWD progression and time to death (TTD) for each individual (the study complied with all IACUC requirements).

In July 2014, seventeen adult *Pisaster ochraceus* were collected by hand from two sites in the Salish Sea, Marine Park, WA (48.719042, -122.516858) (N = 2) and Birch Bay, WA (48.896232, -122.783223) (N = 15), during low tide (Washington State Scientific 111 Collection Permit #15–009 to B.G. Miner). By the time we noticed a seasonal component to the disease in the Salish Sea, suggesting an effect of temperature during this outbreak, most local populations were already impacted by the disease and we were unable to find any sites unaffected by SSWD. Most of the stars we collected were symptomatic, and individuals ranged from no discernible symptoms of SSWD (5 individuals from Birch Bay) to moderate symptoms of infection (widespread aboral white lesions, arm twisting behavior, and loss of turgor; 12 individuals from Birch Bay and Marine Park). Given that most of the individuals showed symptoms of SSWD at each site, we assumed asymptomatic individuals were already infected but not showing signs of SSWD. Sea stars were transported to a temperature controlled laboratory room at Western Washington University.

Specimens were randomly assigned to eight 46cm x 44cm x 21cm acrylic tanks (wall thickness: 0.9cm), two individuals per tank (except for one tank which had three individuals), filled with approximately 38 L of filtered seawater obtained from Shannon Point Marine Center in Anacortes, WA. Five tanks were maintained at 9.0°C by the ambient air temperature of the temperature controlled laboratory room (Bally Engineered Structures, Bally, PA). Nine degrees is similar to winter temperatures in this region (average temperature in January 2014 was approximately 8°C at our site in Marine Park). The remaining three tanks were heated to a mean temperature of 12.1°C, using three 250W aquarium heaters that were currently available for use (forcing an unbalanced design), regulated by D.B.P analog/mechanical thermoregulators (Jumo, East Syracuse, NY) and DynaSense control boxes (Scientific Instruments Inc., Skokie, IL). The 12°C treatment represented summer temperatures though it was several degrees cooler than the average temperature when we collected individuals for the experiment (average temperature at the end of June 2014 was approximately 14°C at our site in Marine Park). Water temperatures in our experimental tanks were measured using Thermochron iButton temperature loggers with a sample interval of 10 minutes placed inside the filter pump of each tank.

All eight tanks were outfitted with AquaClear 20 filter pumps (Hagen, Mansfield, MA), and water was changed weekly in each tank. Water changes consisted of siphoning approximately 19 L of water from each tank followed by replacement with fresh, filtered seawater chilled to approximately 9.0°C obtained from Shannon Point Marine Center. The removed seawater was subsequently sanitized by treatment with a 6% w/v sodium hypochlorite solution at room temperature for approximately 15 minutes prior to disposal to prevent the spread of SSWD. All specimens were maintained on an *ad libitum* diet of live *Mytilus trossulus* Gould 1850, hand collected from Marine Park, Bellingham, WA and added to a tank when more than 50% of *M*. *trossulus* in the tank were consumed.

During the experiment (July through September 2014), all individuals were monitored nearly every day (average = 1.3 d, range = 1–7 d) for signs of SSWD. Individuals were closely inspected for arm twisting behavior, development of *de novo* lesions, spreading of extant lesions, deflation, inability to adhere to the tank walls, arm shedding, and death, and assigned to an infection stage based on observable morbidity (Table 1) [2, 5]. We assigned a stage based on the most matching criteria, with exception of Stage 5. TTD was also recorded for each individual as the number of days each individual survived post-collection. Because some individuals displayed early signs of the disease at the beginning of the experiment, the TTD data do not estimate the time from first sign of the disease to death. However, they are unbiased estimates of the differences between treatments because we randomly assigned individuals to tanks and treatments. Prior to the current SSWD outbreak in 2013, we had reared more than 100 individuals of *P*. *ochraceus* under very similar conditions with 0% mortality.

**Table 1:** Definitions of morbidities of SSWD.

Disease Stage	Morbidity
0	No observable signs
1	Small lesions (< 2mm) on 1 arm or oral disk, lesions cover < 25% of arm or oral disk surface, mild loss of body turgor, mild arm twisting, or mild substratum adhesion deficits
2	Small to medium lesions (< 4mm diameter) on multiple arms or oral disk, lesions cover >50% of arms or oral disk surface, mild to moderate loss of body turgor, or mild to moderate substratum adhesion deficits
3	Small to large lesions (< 6mm in diameter) on multiple arms or oral disk, lesion cover > 75% of arms or oral disk surface, moderate loss of body turgor, moderate to severe substratum adhesion deficits, shedding of 1 arm, or corkscrew arm twisting
4	Small to large lesions (< 6mm in diameter) on 3 or more arms or oral disk surface, lesions cover > 75% of arms or oral disk surface, severe loss of body turgor, severe substratum adhesion deficits, shedding of 2 or more arms, or perforated lesions in which pyloric cecae or gonads protruding from body wall ruptures
5	Death of Individual.

We used a Cox proportional hazard model to analyze the TTD the data [10]. TTD was the response variable and treated as uncensored data because experimental mortality reached 100%, and temperature was the predictor variable. The Cox model was necessary because we lacked experimental data describing the survival distribution of healthy or infected *P*. *ochraceus* in the Salish Sea. An overall *Z*-score was determined for the model, and the significance of temperature as a covariate influencing TTD was determined via a Wald statistic, tested against a *Z* distribution. To account for the nested experimental design (several individuals in each tank), we included tank as a random effect in the model. We used a generalized linear mixed effects model to analyze the morbidity data. Stage was the response variable and time and temperature were the predictor variables. To account for the repeated measures and nesting, we assigned “tank” as a random variable. Because the stage data were ordinal, ranging from 0 to 5 (and integers), we modeled the error with a binomial distribution and a logit link function. R Statistical Software, and the coxme and lme4 packages were used to analyze the data [10, 11].

## Results

**Fig 2:**
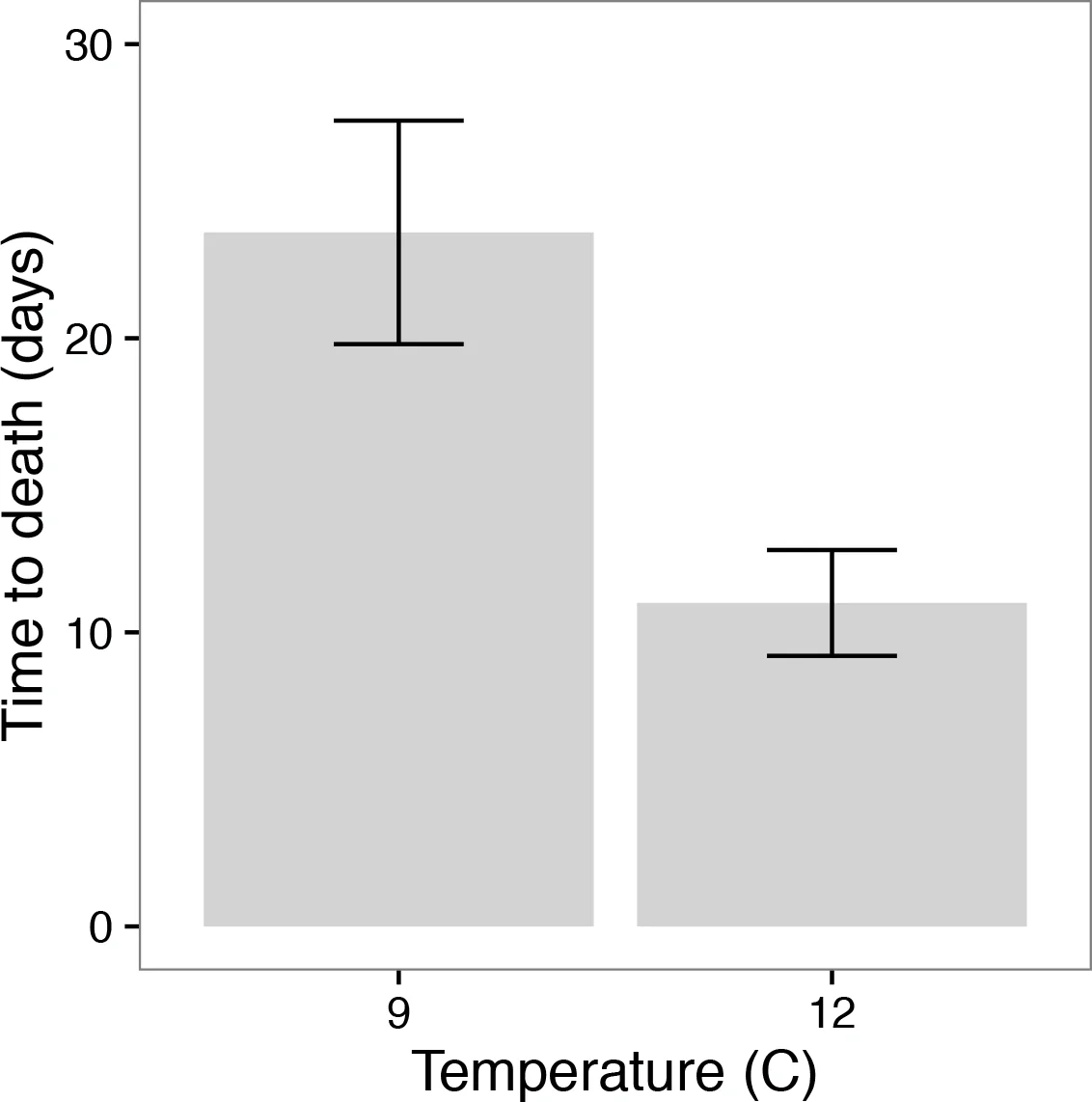
Average time to death in each temperature treatment. Error bars represent ± standard error.

**Fig 3:**
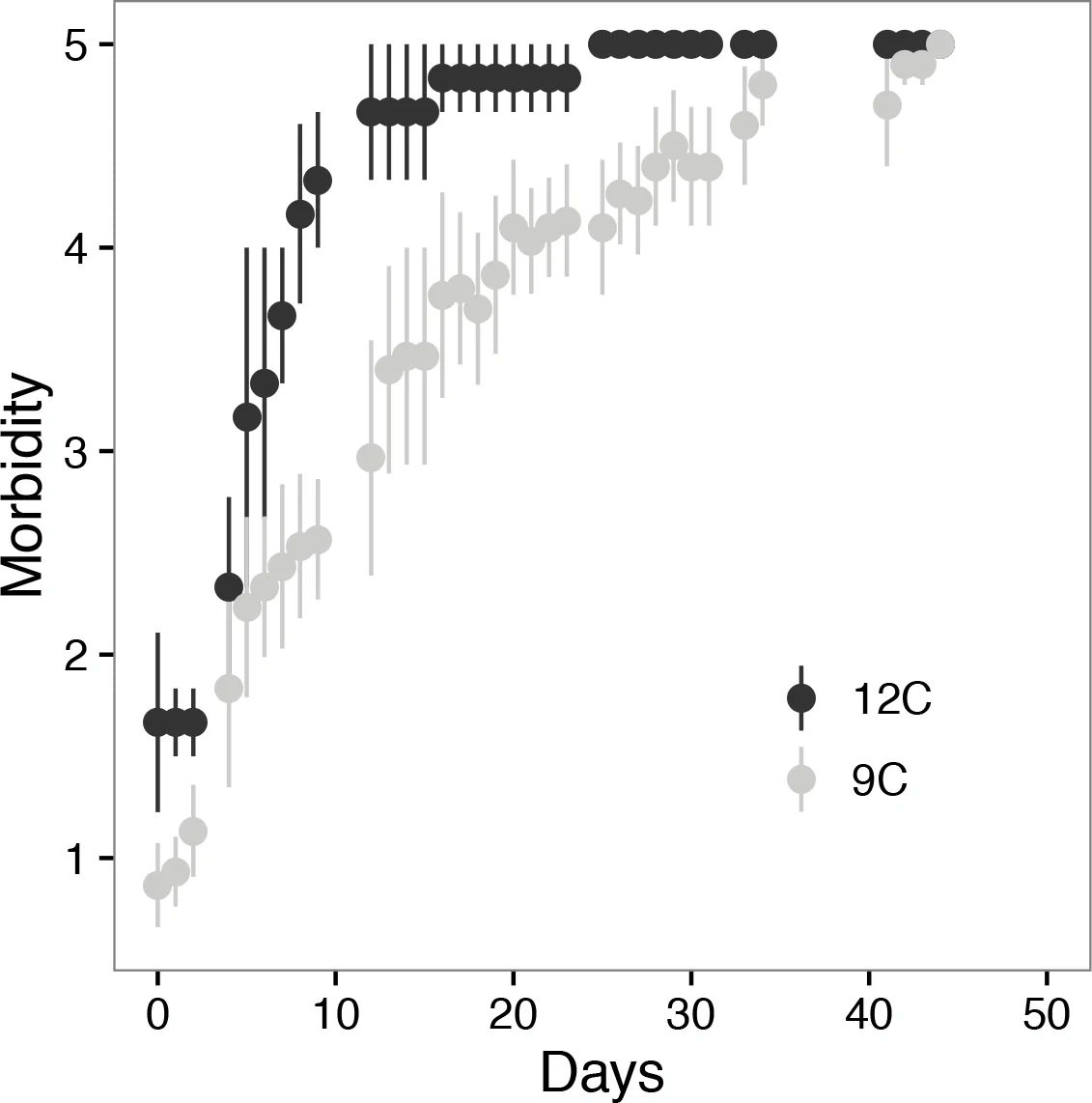
Morbidity vs. time in each temperature treatment. Error bars represent ± standard error.

Temperature significantly affected time to death (β = 1.51 ± 0.6 SE, z = 2.51, P = 0.012; Fig 2). Stars housed at cooler winter temperatures (9°C) lived more than twice as long as individuals housed at ambient summer temperatures (12°C) (Fig 2). However, cooler temperature did not stop, but only slowed the progression of SSWD and delayed death (Fig 3; Table 2). All individuals developed signs of SSWD and died within 44 days of collection. The progression of symptoms in *P*. *ochraceus* was characteristic of SSWD, including: arm twisting, loss of turgor (deflation), *de novo* lesion development, rapid spread of aboral and oral lesions, followed by arm shedding and death (Fig 1). Typically individuals died within 24 hrs of shedding an arm (Fig 3). Similar to field observations, individuals in the 12°C treatment died about a week after signs of SSWD, or SSWD progression were observed (Fig 3). However, dramatic localized epidermal and dermal tissue edema, described as a sign of SSWD in previous studies [2, 3], was not observed in any individuals regardless of treatment level.

**Table 2:** Results of the generalized linear mixed effects model contrasting effects of housing at 9.0°C or 12°C on SSWD-mediated morbidity.

Factor	Estimate	SE	z-value	P-value
Time	0.14	0.0065	21.9	< 0.0001
Temperature	1.35	0.46	2.95	0.0032

Development of *de novo* lesions in individuals initially without signs of SSWD was most often observed in the adambulacral epidermal tissue. Lesions typically spread in distal to proximal and oral to aboral directions. Development or spread of lesions in or to the arm junction of an individual generally proceeded oral to aboral spread. However, aboral to oral spread following development of arm junction lesions was also observed. *De novo* aboral lesions were generally circular or ellipsoid in shape, while most developing oral lesions followed the contours of tissue surrounding the ambulacral groove, only rarely extending into the groove itself. Both oral and aboral lesions were observed to expand as symptoms progressed, such that smaller lesions often “merged” into larger aggregate lesions.

Individuals in both treatments showed severe arm twisting behavior throughout the experiment and this behavior, often in conjunction with individuals losing body turgor and adhesion to the walls of the tanks, was generally observed immediately prior to the development of lesions (Fig 1A). A peculiar type of arm twisting, in which individual arms underwent corkscrew-like contortions with an affected arm curling entirely around on itself multiple times, was observed in several individuals and strongly correlated with arm shedding (Fig 1B).

Corresponding with the time to death, SSWD progressed far more slowly in stars housed at 9.0°C than individuals housed at 12°C. In particular, the spread of extant lesions, as well as development of *de novo* lesions, occurred more slowly in individuals maintained at 9°C than those maintained at 12°C (Fig 3; Table 2). We also observed lesions heal over with apparently healthy epidermal tissue in several stars maintained at 9°C. Furthermore, several stars maintained at 9°C survived weeks with advanced signs of the disease (e.g., severe lesions with perforated body walls and pyloric caeca protruding through the epidermis) (Fig 1C; Fig 3).

## Discussion

In this study, we demonstrate that a decrease in water temperature of 3°C delays SSWD morbidity and mortality in *P*. *ochraceus* collected from the intertidal zone from 2 sites in the Salish Sea. These results support the hypothesis that warmer temperatures *per se*, and not a greater change in temperature, increased the risk to SSWD in previous studies [3, 5, 8]. However, the reduction in temperature did not produce permanent SSWD symptom remission, and all individuals in our experiment eventually died of the disease. Our data are consistent with previous work and field observations demonstrating an increase in susceptibility to infection and progression of SSWD when water is warmer than average, and are the first to experimentally demonstrate that a decrease in water temperature can slow the progression of SSWD in infected individuals during the current outbreak [3, 4, 5, 8, 12]. Though the signs of SSWD did continue to increase in severity at cooler temperatures, some lesions healed during the experiment, and observations of arm-twisting were less frequent compared to individuals in tanks with warmer water. These results might explain the seasonal pattern of SSWD in the Salish Sea, where mortality for sea stars increases during the summer and early fall when waters are warmest.

Previous records of smaller mass mortality events consistently mention observable epidermal and dermal edema as a sign of SSWD; however we did not observe edema in any of our specimens at any stage of infection [3, 5, 12]. We observed the opposite, with mildly and severely infected individuals showing extensive loss of body turgor and flattening prior to significant worsening of symptoms in the former, and prior to arm shedding and death in the latter cases (Fig 1A). Though arm-twisting in stars infected with SSWD was described by Hewson et al. [2], we observed hastened symptom progression in individuals with twisted arms. Furthermore, a specific subset of twisting behaviors (e.g., ‘corkscrew’ arm twisting) were observed to be highly predictive of arm shedding, and to our knowledge this behavioral subset has not yet been described in conjunction with SSWD pathology (Fig 1B). Such differences in the signs and order in which they progress suggest a different or novel underlying pathology from previous wasting mortality events.

We also observed increases in SSWD-mediated morbidity and mortality at a lower ‘high’ temperature treatment level (12°C) than those utilized in previous work (14°C—20°C). In the latter case of a 20°C high temperature this is easily explained by differences between average yearly water temperatures in temperate and tropical ecosystems [3, 5]. The significant decrease in TTD seen in our specimens following housing at 12°C suggests a slightly greater temperature stress sensitivity in Northern Washington State *P*. *ochraceus* populations than seen in conspecifics further West on the coast of Vancouver Island in British Columbia [3]. Regardless of differences in thermal stress sensitivity, our data suggests that increasing sea surface temperatures might have a lasting impact on intertidal populations of this marine invertebrate keystone species in the Pacific Northwest [13].

Sea star associated densovirus is a candidate for the root cause of SSWD but it remains unclear how or if viral infection alone is sufficient for mortality [2]. *Vibrio* microbes have been consistently identified in the remaining tissues of wasted sea stars and other echinoderms following, or shortly preceding death in previous SSWD outbreaks [4, 5, 14]. Though *Vibrio* spp. is commonly found in or on marine invertebrates, these bacteria were isolated from the Crown-of-Thorns Star (*Acanthaster planci*) following an injection of bacterial growth medium that caused symptoms similar to those associated with SSWD, suggesting *Vibrio* might be, at least in part, a cause of SSWD mortality [12, 15]. There is also evidence that increases in water temperature potentiate survival or reproduction of virulent marine *Vibrio* species [16]. Facilitation of high *Vibrio* spp. growth rates via increased temperature, and decreased, but not halted, microbial growth at lower temperatures might thus provide a potential mechanistic explanation for our observation of increased TTD at lower temperatures without complete SSWD remission. Thus, facilitation of *Vibrio* spp. growth via increasing water temperatures in the high and mid intertidal may act as a component of a ‘perfect storm’ of environmental and physiological factors leading to microbial overgrowth and SSWD-associated mortality.
